# Evaluation of the usability and acceptability of the P-STEP® mobile app: feasibility study protocol

**DOI:** 10.1186/s40814-024-01546-9

**Published:** 2024-09-13

**Authors:** Hannah Worboys, Laura Gray, Sarah Anthony, Rachel Hobson, Tim Lucas, Andre Ng

**Affiliations:** 1 Department of Population Health Sciences, University of Leicester, Leicester, United Kingdom; 2 Department of Cardiovascular Sciences, University of Leicester, Leicester, United Kingdom

**Keywords:** Environment, Pollution, Physical Exercise

## Abstract

**Background:**

The new P-STEP® (Personalised Space Technology Exercise Platform) app is designed to bring together tailored exercise guidance and up-to-date air quality information. The app allows individuals to plan outdoor exercise walking routes while minimising their exposure to air pollution. Individuals with chronic long-term conditions, particularly respiratory and cardiovascular conditions, can use the app in order to minimise the risk of their symptoms being exacerbated by pollution, while still gaining the benefits of outdoor exercise.

**Methods:**

This study will measure the usability and acceptability of the P-STEP® app. The study will take the form of a single-arm 12-week app pilot study based in Leicestershire, United Kingdom (UK). We will recruit a maximum of 380 participants from an existing cohort study to pilot the app for 12 weeks. Questionnaire data will be collected at three timepoints, baseline, 6 weeks and 12 weeks. The primary outcome is the System Usability Scale at 12 weeks. Secondary outcomes include the User Engagement Scale Short Form, SF-12, Recent Physical Activity Questionnaire (RPAQ), bespoke, app specific usability questions, and feasibility outcomes. Additional data collected includes participant demographic information, technology self-efficacy and adverse events. Weekly anonymised usage data from the app will also be collected by the app team and analysed separately to complement the questionnaire data.

**Discussion:**

This study will help us better understand the feasibility and acceptability of using the P-STEP® in the community. The results will also help inform future studies.

**Ethics and dissemination:**

This study has received ethical approval from the South West Frenchay Research Ethics (23/SW/0060) Committee. There is no need for further approval from the Health Research Authority as the study is not taking place in the NHS. The ClinicalTrials.gov ID number is NCT05830318.

**Supplementary Information:**

The online version contains supplementary material available at 10.1186/s40814-024-01546-9.

## Introduction

### Background

Lifestyle changes such as increasing physical activity levels can greatly reduce the risk of mortality for people with long-term conditions (LTCs) [1]. Exercise is crucial to health, and it is recommended that an individual does a minimum of 150 minutes moderate intensity exercise per week [2]. Despite the benefits of regular physical activity, many patients living with LTCs, such as asthma, COPD and heart disease, remain inactive and do not meet current guidelines [3, 4].

Air pollution is an important factor for patients with LTCs and can severely impact those suffering from cardiac and respiratory diseases [5, 6]. There is a risk of exacerbating symptoms when exercising outdoors, if air quality is poor, which may be leading to physical inactivity in some patients. Many patients live in fear of worsening symptoms and therefore minimise their exposure to air pollution by remaining indoors [1].

Mobile phone applications (apps) are becoming increasingly used to help to support those with LTCs, including advice on behavioural change such as increasing activity levels. Many apps also exist to provide information on air quality. To date, no app has been developed to combine these two related issues. The Personalised Space Technology Exercise Platform (P-STEP®) app brings together tailored exercise guidance, taking into account an individual’s LTC(s), while also providing up-to-date information on air quality. The app allows individuals to plan exercise routes in order to minimise their exposure to air pollution, by using the information to advise avoiding higher polluted areas.

### Research question

The purpose of this study is to assess the usability and acceptance of the P-STEP® app, by allowing participants with specific LTCs to pilot the app for a 12-week period. We will also assess the feasibility of study design features and outcome measures, which will help inform a future clinical trial.

## Methods

### Design

The study design will take the form of a single arm 12-week app pilot study based in Leicestershire, United Kingdom (UK). Questionnaire data will be collected at three timepoints, baseline, 6 weeks and 12 weeks. Weekly anonymised usage data from the app will also be collected by the app team. We will recruit a maximum of 380 participants to pilot the app for 12 weeks. This study has been approved by the South West Frenchay Research Ethics Committee (REC), who confirmed that there is no need for further Health Research Authority (HRA) approval. The adapted SPIRIT (Standard Protocol Items: Recommendations for Interventional Trials) checklist for reporting the protocol of a pilot study has been included in supplementary information Table S2 [7–9]. The SPIRIT figure, which demonstrates the schedule of enrolment, interventions, and assessments, is included in Figure S1 [7].

### Screening and recruitment

Participants will be recruited from the Extended Cohort for E-health, Environment and DNA (EXCEED) cohort study. EXCEED (REC ref 13/EM/0226) is a longitudinal population-based cohort study that facilitates the investigation of genetic, environmental and lifestyle-related determinants of a broad range of diseases and of multiple long-term conditions [10]. EXCEED participants have consented to be contacted about participating in other research studies. We are using the EXCEED cohort because we are focusing on recruiting participants with particular LTCs, and EXCEED will give us access to these participants. For example, in 2019, the numbers of participants in EXCEED diagnosed with at least one of the conditions we require were as follows: asthma (*n* = 1138), heart disease (*n* = 393), COPD (*n* = 301), diabetes (*n* = 826) and heart failure (*n* = 107) [5]. The EXCEED team will identify participants and invite them to join the study, based on the following criteria:

- Adult ≥ 18 years
- Live in Leicestershire
- Diagnosed with at least one of the following conditions*:
  o Asthma
  o Chronic obstructive pulmonary disease (COPD)
  o Interstitial lung disease (ILD)
  o Coronary heart disease (CHD)
  o Heart failure (HF)
  o Type 2 diabetes
- Do not have dementia, learning disability, severe mental health disorders (other than depression or anxiety) or epilepsy
- Are not receiving palliative care

*This criterion will be relaxed if recruitment is slower than anticipated.

The EXCEED research team will email potential participants who meet the above criteria with information about the P-STEP® pilot study and give them an individual link to a closed webpage. Potential participants will have the opportunity to read more about the study and register their interest through an MS Forms. Those who register their interest in the study will then be screened as to whether they meet the following additional inclusion criteria:

- Android smartphone which has access to the internet
- Can walk outside for a minimum of 5 min without feeling unsteady
- Available to pilot the app from June 2023 to January 2024
- Able to give informed consent to participate in the evaluation

And will be excluded if:

- English language restriction (it is made clear that documents and app are only available in English, and participants should only register if confident with this.)
- Advised not to do exercise by a health care professional, in the last 12 months
- Access to an iOS smartphone only
- Diagnosed with dementia, learning disability, severe mental health disorders (other than depression or anxiety), epilepsy
- Receiving palliative care
- Pregnant
- Chest pain at rest
- Unsteady when standing or walking
- Current cancer patient
- Part of the P-STEP® User Engagement group

### Study duration, data collection and follow-up

Participants that meet the inclusion criteria will be invited to enrol on to the study; this includes going through a process of formal informed consent. The participant information sheet and consent form will be emailed to participants via REDCap, a secure web platform for collecting and managing online survey data [11, 12]. A copy of the consent form has been included in the supplementary information Figure S2. After participants have filled in the consent form, it will be checked and signed by a member of the research team. The participant will then complete the baseline questionnaire, which will also be sent via the REDCap system. The baseline questionnaire is a shortened version of the follow-up questionnaire that asks only about participant demographics and characteristics, quality of life and physical activity levels. Questions about the app are then included at follow-up. After returning the baseline questionnaire, participants will then be texted the link to download the app.

After participants have been given access, they will pilot the app for 12 weeks. Questionnaires will be sent out to participants for them to self-administer and complete at 6- and 12-week post baseline, via REDCap. Questionnaires administered and data collected will be the same at both the 6- and 12-week follow-ups. Separate from the questionnaires, objective usage data will be automatically collected by the app and returned to the app development team on a weekly basis. These data are anonymous, and it is not possible to link this information directly to a participant. Table 1 displays the objective usage data we will collect for this study.

**Table 1:** Usage data

Usage information	Rationale/use
Number of downloads	Number of downloads will give us an idea of interest and the feasibility of administering the app. Successful downloads means the users have successfully clicked on the link and downloaded the app
Number of logins	At the beginning stages of the study, we can compare this with number of downloads. Later in the study it will give us an idea of whether people log-out and come back to log-in or stay logged in the duration of the study
Number of walks recorded	Number of physical activities recorded in a week
Clicks on feature	Will give us an idea on how much each feature is used across participants
Time spent on feature	Will give us an idea on how much each feature is used across participants
Clicks on links to external sites	Will give us an idea on how much each links to external sources are used across participants
Notifications delivered	Help us to identify how much notifications are enabled, how many clicks from notifications we get, can identify if there is a surge in use after a notification has been received
Number of log outs	Idea on behaviours such as whether users log in/log out, stay logged in, stay logged out
Number of app uninstalls	Idea as to how many users do not like the app, therefore delete during the study period
Device/model the app has been installed on	Feasibility of app on different devices

### Patient and public involvement (PPI)

Members from the University Hospitals Leicester NHS Trust Lifestyle PPI group and members from the EXCEED cohort (described below) have been involved in the design of this pilot study. The PPI for this study included designing and reviewing the study documents such as the register your interest form, participant information sheet, informed consent form, baseline and follow-up questionnaires. PPI members were also included in reviewing the participant pathway to identify ways to minimise the burden to participants.

## Peer review for ethics approval

The protocol for this study was reviewed by two individuals, independent from the project and study team, prior to gaining Ethics approval. Both reviewers mentioned the need to consider multiple mitigation strategies to prevent potential high dropout. Multiple strategies were incorporated into the study, including giving participants the opportunity to be supported by a member of the study team when downloading the app, and setting up a helpline to encourage users to use if they are having any trouble with accessing/using the app.

Reviewer 2 commented on how many participants we are expected to recruit from each of the long-term condition (LTC) groups, and how many with a single LTC vs multiple LTCs. We will not be stratifying by LTC at the recruitment stage. This is because for feasibility reasons, we would like to see which LTC groups come forward and volunteer to pilot the app. As well as this, we expect a large proportion of patients to have multiple LTCs, given the nature of the EXCEED cohort, and will not be excluding participants for this.

Reviewer 2 requested a more detailed definition of “Usability”. This has been updated.

Reviewer 2 asked for the reason we are only recruiting from the EXCEED cohort for participants in this study. The reason for this is that we wanted to ensure that we are recruiting participants with the six LTCs that we are interested in. The EXCEED cohort study group gives us direct access to these participant groups to help with recruitment.

Reviewer 2 commented on the long list of exclusion criteria; however, for any study involving physical activity, be it interventional or guidance, detailed exclusion criteria is required in order to ensure the maximum safety for participants.

## Data analysis

### Bias

Participants will be asked to provide informed consent and will be given detailed information on the structure of the study to ensure a full understanding.

To avoid user bias, we will exclude participants who are part of the P-STEP® User Engagement group as they have been involved in the design of both the app and the study. All participants that complete the baseline questionnaire will be included in the study and analysis.

Participants are being recruited only from the EXCEED cohort. We acknowledge that by recruiting only through EXCEED, we are recruiting individuals who have signed up to be part of a cohort study (and therefore, a part of future research) which may not necessarily represent the general population.

We acknowledge that including only English-speaking participants may exclude certain groups; however, to date, the app has only been developed in English, and as this is a usability and acceptability study, it felt that this restriction is necessary.

## Risks

Questions asked to participants will avoid areas of cultural sensitivity and will be focused around the participants’ experience with the app. As we are also collecting some demographic information, data will be anonymised and handled in accordance with data protection procedures at the University of Leicester. Participants will be required to download and use the app onto their mobile phone, which they do so at their own risk after accepting the app terms and conditions. Use of the app may consume mobile data. It is recommended to participants for the app to be downloaded while connected to WIFI to avoid the use of mobile data. Participants will be provided with vouchers to compensate for any additional data usage incurred or purchased upon provision of receipts, up to a total of £20.

### Sample size

As this is a pilot study, a formal power calculation has not been undertaken. Initial searches by the EXCEED team to estimate the population size for this study, given the first stage of inclusion criteria, is 1525. Given the second stage of inclusion criteria, we estimate a maximum interest rate of 380 (based on 50% passing the eligibility criteria and then only 50% of them having Android phones). We will therefore recruit up to a maximum number of 380 participants. While this sample size is larger than other pilot studies, it has been reported in previous studies that while testing the usability of apps, there are higher levels of dropouts (up to 80%), than other types of pilot studies [13]. Recruiting 380 participants with an 80% dropout rate would result in 76 participants completing the study. Previous studies [14, 15] in the space of assessing usability with the System Usability Scale have reached conclusions with a sample size of 20. General guidance [16–18] on feasibility studies suggests that a sample size of 30–50 participants is an adequate number to make conclusions for both the usability and feasibility outcomes. We believe that this will be an adequate group to make conclusions on the usability of the app, as well as information to inform a future trial. Anyone that registers their interest after the deadline, or when the maximum is reached, will be thanked for their interest but told that recruitment is closed. The additional registrations of interest will be included in the feasibility outcomes.

### Statistical analysis

Baseline characteristics will be summarised using mean and standard deviation (SD) or median and interquartile range (IQR) for continuous variables and count and percentage/proportion for categorical. Each questionnaire will be scored and analysed using the methods described by the developers of the questionnaires. The primary outcome measure for this study is the System Usability Scale (SUS) at 12 weeks. The mean and standard deviation of the SUS, usability and user engagement scores will be reported at 6 and 12 weeks. A breakdown of responses to the SUS questionnaire will be tabulated. The SF-12 and RPAQ will be collected at baseline, 6 and 12 weeks. An indication of changes over time will be reported using a mixed effects model. Feasibility outcomes will be reported as a mean and standard deviation or count and percentage where appropriate.

All statistical analyses will be performed in Stata v17/BE.

### Outcomes

#### Primary outcome measure

The primary outcome measure for this study is the System Usability Scale (SUS) at 12 weeks. The SUS is a validated and popular instrument for measuring perceived usability [8]. There are 10 items in total, 5 with a positive tone and 5 with a negative tone. The responses range from strongly disagree to strongly agree. The original questionnaire asks about the “system”; however, it is an acceptable practice to replace system with a relevant term such as website, or app; therefore, this study replaces the word system with app. The SUS responses will be scored using the methods described by the developers of the questionnaire [19]. Each item’s score contribution ranges from 0 to 4. For items 1, 3, 5, 7 and 9, the score contribution is the scale position minus 1. For items 2, 4, 6, 8 and 10, the contribution is 5 minus the scale position. The score is then multiplied by 2.5 to obtain the overall value of the SUS on a scale of 0–100. General guidance suggests a SUS score 68 and above considers the usability score to be above average and anything less below average [19]. The mean and standard deviation of the usability scores will be reported at 6 and 12 weeks.

### Secondary outcome measures

#### Usability questions (P-STEP® specific)

Nine additional usability questions, detailed in Table S1, that relate specifically to the features of the P-STEP® app, will be asked at 6 and 12 weeks. These questions have had input in from our PPI group, and through a process of iteration, we finalised nine questions on a Likert scale of strongly agree to strongly disagree. Individual items in the questionnaire will be tabulated with percentages. For example, 77% of participants strongly agreed that the app was suitable for their age group. The mean and standard deviation of the usability scores will be reported at 6 and 12 weeks.

#### User Engagement Scale-Short Form (UES-SF)

The User Engagement Scale-Short Form (UES-SF) is a reliable and valid questionnaire containing 12 items that measure user engagement [8]. There are 12 items which are categorised into: focused attention, perceived usability, aesthetic appeal and reward. The UES-SF responses will be scored using the methods described by the developers of the questionnaire [8]. An overall user engagement is calculated by converting the score to a numeric value of 1–5 (strongly disagree equals 1, strongly agree equals 5) and taking the mean. The mean and standard deviation of the UES-SF scores will be reported at 6 and 12 weeks.

#### Quality of Life

The Short Form 12-Item Health Survey [9, 10] (SF-12) is a health-related quality of life tool that measures functional health and well-being from the participant’s perspective. It assesses eight domains: physical functioning, physical role, pain, general health, vitality, social functioning, social role and mental health. SF-12v2 responses will be scored and interpreted using the SF-12v2 user guide [9]. The eight domains will generate two summary scores: physical component score (PCS) and mental component score (MCS). Scores for the PCS and MCS range from 0 to 100, and a higher score indicates a higher quality of life. The participant’s quality of life will be calculated at baseline, 6 weeks and 12 weeks. An indication of change in quality of life over time will be reported using a mixed effects model.

#### Recent Physical Activity Questionnaire (RPAQ)

The Recent Physical Activity Questionnaire [11] (RPAQ) is designed to find out about a participant’s physical activity in their everyday life. The RPAQ will be scored and interpreted using the scoring guidelines published by the authors. The participant’s RPAQ score will be calculated at baseline, 6 weeks and 12 weeks. An indication of change in physical activity over time will be reported using a mixed effects model.

#### Usage data and feasibility outcomes

Usage data will be extracted weekly by the app team. This information will give us an indication of the usage of the features of the P-STEP® app which will be used to inform the feasibility outcomes. The data extracted will be a summary of all participants; however, this information is anonymous and therefore cannot be linked back to participants. Feasibility outcomes will be reported as count, mean, proportions or length of activities in minutes. The feasibility outcomes that will be reported are presented in Table 2. These outcomes will assess the feasibility of using certain study design features and outcome measures which will help plan a future trial for effectiveness.

**Table 2:** Feasibility outcomes

Feasibility outcome	Measure
Interest	Percentage of those eligible who register their interest
Eligibility	Percentage of participants who register their interest that pass eligibility screening and enrol
Enrolment	Percentage of those who pass eligibility who then go on to enrol on the study
Feasibility of administering the app	Percentage that successfully download the app Percentage that are on boarded vs download independently
Acceptability of the app	Percentage of participants who enrol and complete the 12-week study
Acceptability of outcome measures	Percentage of participants who complete baseline, 6 and 12 week follow-up
Acceptability of online questionnaires	Percentage that require help to complete online questionnaires Percentage of withdrawals that give method of data collection as reason
The proportion of screened participants ineligible and reasons for ineligibility	Number/proportion of participants screened and deemed ineligible

The feasibility of recruiting through a cohort study (EXCEED) will be assessed through the interest outcome in the feasibility table. This will give an indication of whether recruitment through a cohort study for a future study would be feasible or if alternate strategies, such as recruitment through GP practices, would need to be considered. We will also assess the interest in certain groups (e.g. with each of the conditions), and this will further inform how recruitment is structured moving forward to ensure specific groups are targeted if necessary.

### Adverse events and serious adverse events

Adverse events (AEs) and serious adverse events (SAEs) will be recorded in line with University of Leicester policy. Participants will be asked some additional health questions at week 6 and 12 that will allow us to report AEs/SAEs.

### Long-term clinical outcomes

Finally, we will gain consent from the P-STEP® participants for the data collected in this study to be transferred to the EXCEED study team. This will enable linkage to electronic health records (EHR) in the future and allows the EXCEED study team to analyse long-term outcomes such as mortality and cardiovascular events. The primary data collected in this study, together with EHR records, will allow the monitoring of long-term clinical outcomes.

### Success criteria of the study

We expect to see above average usability scores and medium engagement, scoring at least 68 on the SUS and bespoke usability questionnaires and at least a 3 out of 5 on all engagement domains for the User Engagement Scale Short Form. Questions worded negatively will be transposed such that 3 out of 5 on the User Engagement Scale has the same meaning for each response. The free text fields will allow participants to comment on the functionality of the app which can be taken forward to future iterations, prior to a full-scale trial. For the feasibility outcomes, we deem rates of over 85% as acceptable and will take forward those aspects of study design when planning a future trial. Anything less than this will be reviewed and addressed or removed as appropriate during the design of a future trial.

### Missing data

Participants that drop out will be encouraged to still complete the questionnaires. Participants that drop out between 6 and 12 weeks will be analysed using the available data (6-week responses). Where scoring guidelines from the above questionnaires have mentioned how to deal with missing data, these guidelines will be followed.

## Data management

### Data collection and source data verification

Participants will receive a unique link to the questionnaires via email at baseline, 6 weeks and 12 weeks and will be given a deadline to fill in the questionnaires. Copies of the fully signed consent form will be provided to the participant via email. A Data Management Plan will be created by the Project Management Group with specific details on data handling and record keeping. The usage dataset generated by the app itself will be stored securely. This data is collected weekly and summarizes users’ interactions with the app.

### Data protection and patient confidentiality

Personal data included in study-related databases shall be treated in confidence and in compliance with ICH-GCP, the UK Policy Framework for Health and Social Care, the Data Protection Act 2018 (DPA 2018) and the EU General Data Protection Regulation (GDPR).

### Data access and storage

All study documentation will be retained in a secure location during the conduct of the study. Personal identifiable data will be retained by the study team for a maximum of 12 months following the end of the study, after which it will be destroyed. Personal identifiable details such as names and contact details will be retained on a password protected database until required and then destroyed. All electronic data will be stored on secure network systems, to which only the relevant study personnel will have access. The dataset will be anonymised and stored securely.

## Supplementary Information

Supplementary Material 1: Table S1: Bespoke usability questionnaires. Table S2: Adapted SPIRIT checklist for reporting the protocol of a pilot study.

Supplementary Material 2: Figure S1: Schedule of enrolment, interventions and assessments. Figure S2: Informed Consent Form. 

## Data Availability

Data is confidential and cannot be shared.
