# Supplementary material for: Evaluation of the usability and acceptability of the P-STEP® mobile app: feasibility study protocol
Source: Pilot Feasibility Stud. 2024 Sep 13;10:120. doi: 10.1186/s40814-024-01546-9 (PMC11395691; doi:10.1186/s40814-024-01546-9)

**Figure S1: Schedule of enrolment, interventions and assessments**

| Timepoint | Enrolment | Allocation | 12-week study duration | Close-out |
| --- | --- | --- | --- | --- |
| Register interest | X |  |  |  |
| Eligibility screen | X |  |  |  |
| Informed consent | X |  |  |  |
| Given access to app |  | X |  |  |
| Baseline assessment |  |  | X |  |
| 6 week follow up |  |  | X |  |
| 12 week follow up |  |  | X | X |

**Figure S2: Informed Consent Form**


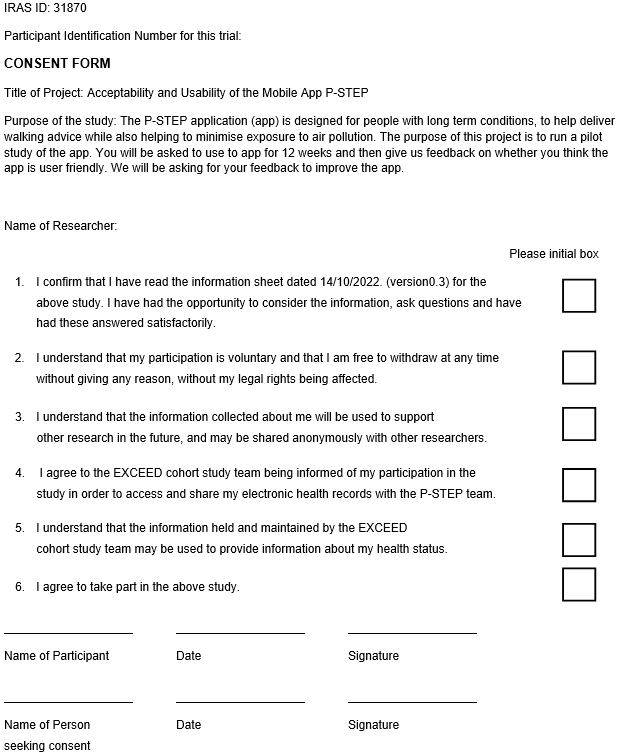

Supplement: Supplementary file 2 — Supplementary Material 2: Figure S1: Schedule of enrolment, interventions and assessments. Figure S2: Informed Consent Form. [file 40814_2024_1546_MOESM2_ESM.docx]
